# Novel Analgesics with Peripheral Targets

**DOI:** 10.1007/s13311-020-00937-z

**Published:** 2020-10-15

**Authors:** Cosmin I. Ciotu, Michael J. M. Fischer

**Affiliations:** Center of Physiology and Pharmacology, Medical University of Vienna, Schwarzspanierstrasse 17, 1090 Vienna, Austria

**Keywords:** Inflammation, cytokine, pain, receptor, sensory neuron.

## Abstract

A limited number of peripheral targets generate pain. Inflammatory mediators can sensitize these. The review addresses targets acting exclusively or predominantly on sensory neurons, mediators involved in inflammation targeting sensory neurons, and mediators involved in a more general inflammatory process, of which an analgesic effect secondary to an anti-inflammatory effect can be expected. Different approaches to address these systems are discussed, including scavenging proinflammatory mediators, applying anti-inflammatory mediators, and inhibiting proinflammatory or facilitating anti-inflammatory receptors. New approaches are contrasted to established ones; the current stage of progress is mentioned, in particular considering whether there is data from a molecular and cellular level, from animals, or from human trials, including an early stage after a market release. An overview of publication activity is presented, considering a IuPhar/BPS-curated list of targets with restriction to pain-related publications, which was also used to identify topics.

**Electronic supplementary material:**

The online version of this article (10.1007/s13311-020-00937-z.) contains supplementary material, which is available to authorized users.

## Introduction

**Fig. 1:**
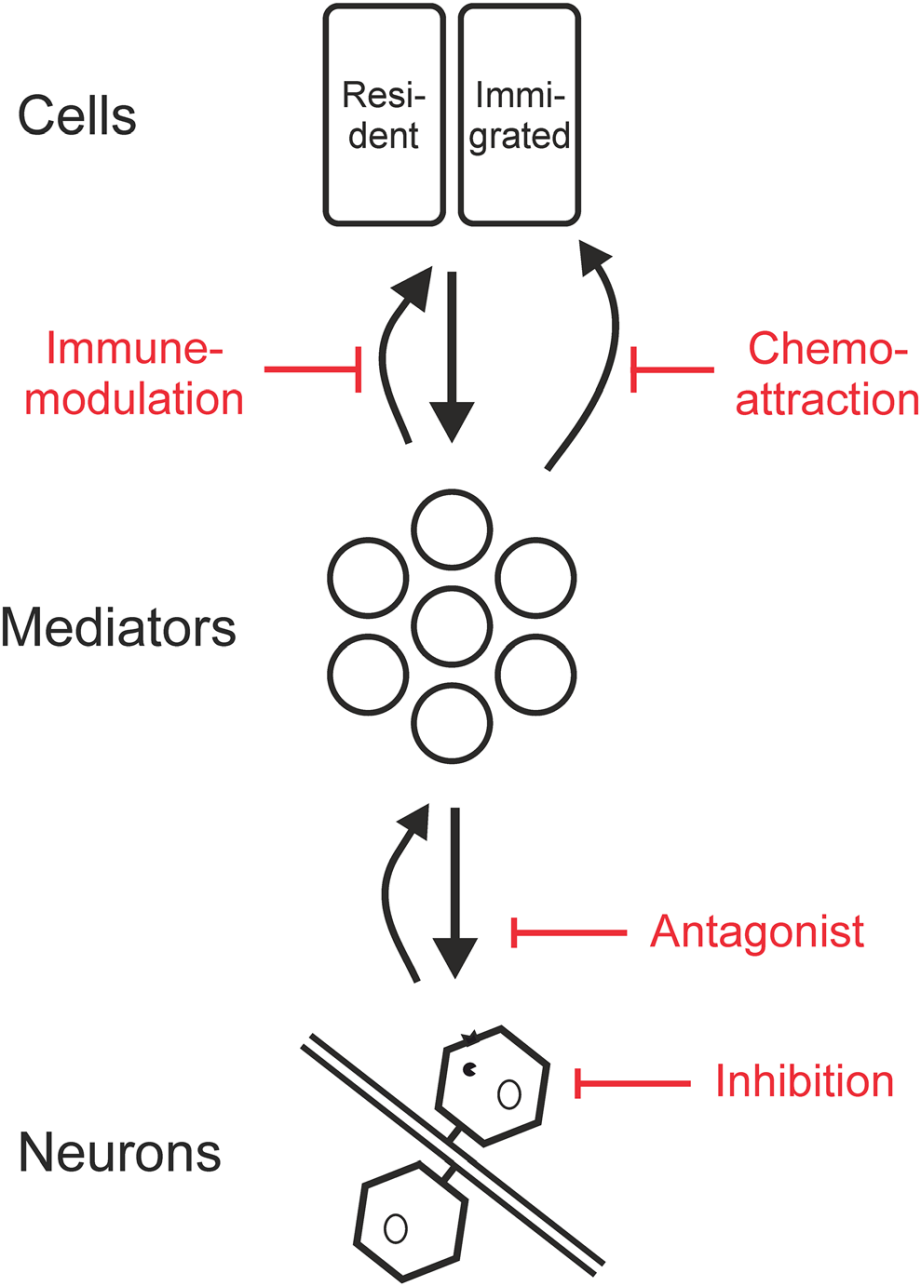
Pathophysiological concept, also serving as outline for the review. Neuronal and non-neuronal cells were separated to provide a schematic for therapeutic approaches. These are discussed in the review, first considering direct inhibition of the neurons and then antagonizing mediators acting on neurons. For modulation of the immune response, separated in local modulation and chemoattraction as well as addressing systemic inflammation, the reader is referred to the reviews of the respective topic. In the neuron, a receptor and an enzyme are visualized as targets

Analgesia is a medically important issue, with a large body of primary and review literature. Therefore, this review starts with an outline of the aim and the approach to the topic. The scope is to discuss novel analgesic approaches [1–3], including all stages of preclinical evidence and, in particular, approaches in the clinical trial phase. Established pain medications are mentioned, where they serve to discuss concepts or serve as a benchmark for novel approaches. A concept figure illustrates a general view on peripheral nociception and analgesic approaches (Fig. 1). Within this framework, the review discusses bottom-up direct effects predominantly or exclusively on the neuron, then inflammatory mediators with at least partial action on sensory neurons. We accept that this structure also has disadvantages. We have tried to avoid a splitting of ligands from their targets where possible, and also refer to other approaches to structure the topic [4, 5]. For mechanisms which are in summary anti-inflammatory, analgesia can be expected as a collateral effect. The other targets include resident and migrating cells. Resident cells include mast cells, macrophages, neutrophils, and Schwann cells. For this more general immunomodulation and systemic anti-inflammation, the reader is referred to the respective literature [6, 7]. Within neuronal targets, there might be some imprecision due to findings from afferent ganglia, which contain other cell types than neurons and a more detailed allocation to the cell type is unknown. Antipruritic approaches have a considerable overlap with analgesia; therefore, a conceptually similar view can be found in the respective literature [8].

**Fig. 2:**
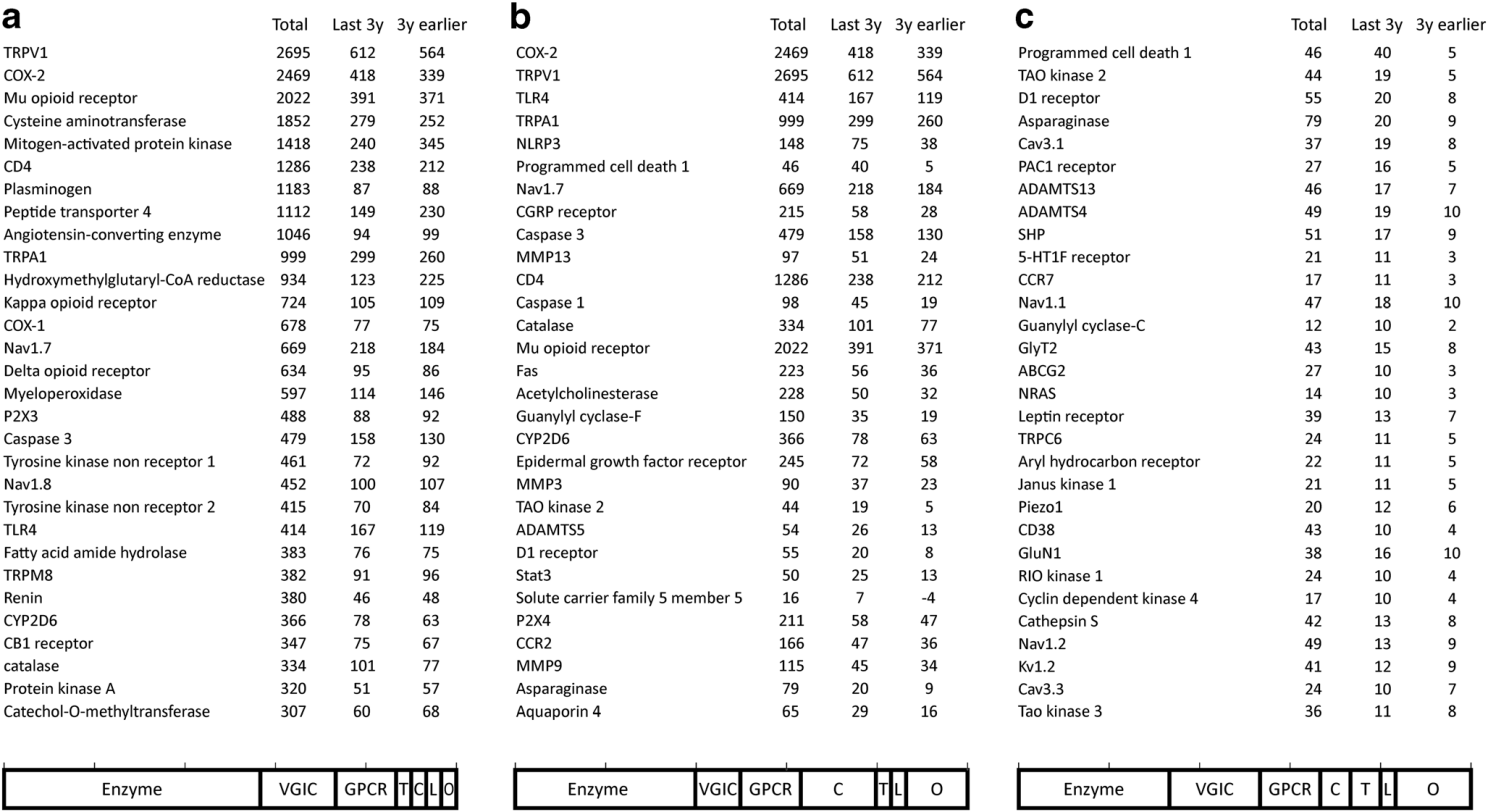
Pain targets identified by Pubmed scraping. The search was performed in April 2020. (**a**) “Large topics,” assessed by total publications without time restriction to indicate overall interest in this topic, sorted by the Total column. (**b**) “Rising topics,” sorted by the difference between the publications within the last 3 years minus the 3 years before. (**c**) “Novel topics,” considering targets exceeding 10 publications within the last 3 years but not in the 3 years earlier; the delta was used for sorting. The cumulative bar chart at the bottom shows the distribution by target type. VGIC = voltage-gated ion channel, GPCR = G protein-coupled receptor, T = transporter, C = catalytic receptor, L = ligand-gated ion channel, O = other

This review focuses on recently published approaches, but ways to identify new targets should also be mentioned [9]. For identifying published topics, Pubmed was queried utilizing the NCBI E-Utilities [10]. As search terms, “pain” and the potential targets in inverted commas were entered. For the latter, the “targets and family” list of the IuPhar/BPS database [11] was used. As a caveat, searches by human gene nomenclature (HGNC) name, rat genome database (RGD) name, or the respective short forms provide a different rate of false positives and negatives, so the respective search term list required manual optimization. How to identify trends in these data? This can be addressed by complex strategies [12]; nevertheless, an easily comprehensible approach, considering rising publications per time, was preferred here. As suggested [13], a 3-year interval was chosen. The search was conducted in April 2020. A comparison of the last 3 years in contrast to the prior 3 years provides a trend of a respective topic. Three indicators for the targets are presented (Fig. 2): a) Already “Large topics,” assessed by the total number of publications, b) “Rising topics,” calculated as publications within the last 3 years minus the 3 years before, and c) “Novel topics,” considering only targets which have more than 10 publications within the last 3 years but no more than 10 publications in the 3 years before. The overall content is organized as outlined by Fig. 1, within this structure sorted by target type, e.g., GPCRs, ion channels, enzymes. Figure 2 was also used to pick topics.

## Effects Predominantly or Exclusively on Sensory Neurons

### GPCRs in Sensory Neurons

GPCRs form the largest receptor superfamily, with 1265 sequenced members in humans [14]. They play crucial roles in inflammation, with abundant expression in immune cells (regulating migration, accumulation at the inflammation site and phagocytosis), endothelial cells (regulating permeability), and nervous tissue, to name a few. As a brief reference to the above-mentioned inflammatory mediators and their GPCR targets, PAR1–4, S1P1, LPAR1–6, prostaglandin receptors, bradykinin receptors, and NK1 are among the most relevant families for inflammatory hyperalgesia. Ligands binding to these GPCRs modulate the three main signaling pathways of the G-alpha subunits Gi, Gs, and Gq, which have typical effects. For G-beta/gamma targets, the reader is referred to a review [15]. Gi couples inhibiting, Gs stimulating to adenylate cyclase, and prostaglandin and opioid receptor effects discussed below might be seen pro toto. Protein kinase A downstream of Gs has been shown to sensitize a variety of ion channels involved in nociception, similar to protein kinase C via Gq and Phospholipase C (PLC) [16]. Downstream of PLC, the intracellular signaling cascades diverge, and include protein kinase C (PKC) and calcium admission. Most of the inflammation pathways (leading to short-term hyperalgesia, in any case) converge onto these mechanisms.

### Opioid Receptors

Opioids are an essential pillar of analgesic therapy. This will not change any time soon, opioid crisis or not, and a top spot in publications reflects that importance. Opioid analgesia in inflammation is the result of combined central and peripheral mechanisms [17, 18], and following the scope of this review, we briefly expand on the latter. Peripheral sensory neurons express the μ-, δ-, and κ-opioid receptors [19, 20]. The opioid receptors are Gi-coupled and the downstream mechanisms apply to other Gi-coupled GRCRs on sensory neurons. Endogenous agonists met- and leu-enkephalin are broken down by neutral endopeptidase and aminopeptidase N. Single inhibition of these enzymes was not analgesic in humans [21]. On the other hand, inhibition of both enzymes has been successfully tested in animals [22], but only with intrathecal application in humans [23]. Progression of enkephalin inhibitors has been discussed [24], and there is sparse but ongoing development for analgesia [25]. Pharmacological intervention with opioids leads to analgesia via a reduction in neuronal excitability [26]. Thereby, opioid receptor activation also reduces neurogenic inflammation, e.g., by limiting calcitonin gene-related peptide (CGRP) and substance P release. In inflammatory conditions, opioid receptors are upregulated in the dorsal root ganglia (DRG) and their trafficking to the nerve endings seems to increase [27]. Moreover, with a prolonged inflammatory event, peripheral receptors seem to account for the majority of the analgesic effect [28]. Peripherally restricted μ-opioid receptor antagonists methylnaltrexone, alvimopan, and naloxegol could allow to reduce dose-limiting side effects of blood–brain barrier–permeable agonists, e.g., constipation [29]. A similar strategy should work for developing kappa-receptor agonists. Efforts have resulted in several compounds including ADL 10-0101 (discontinued after phase II) [30], topical loperamide for pain due to repeated finger lancing [31], and oxycodegol (NKTR-181, after completed phase III not commercialized due to FDA advisory board vote) [32, 33]. 

Novel κ-receptor agonists include CR665 (not further developed after phase II), which showed efficacy in alleviating symptoms of visceral pain [34]: JT09 designed for oral administration [35], difelikefalin (CR845, phase III completed 2020) [36], and TRK-820 (not further developed after phase III) [37]—investigated in pruritus. δ-opioid receptor agonists include GIC-1001 (phase II started in 2013) [38, 39]. In summary, there is a lot of activity to expand the opioid-based therapeutic options.

### Prostaglandin Receptors

These GPCRs include DP_1_ (PGD_2_ receptor), EP_1_–EP_4_ (PGE receptors), FP (PGF receptor), IP (PGI receptor), and TP (TXA receptor). It has been shown that murine peripheral nociceptive neurons express IP, EP_1_, EP_3_, and EP_4_ mRNAs [40]. The respective mediators and the generating enzymes are discussed in the second chapter. The sensitizing effects of PGE_2_ and PGI are well known [41], characterized by a sensitization of TRPV1 channels in DRG neurons downstream of EP_1_ and IP receptors through PKA and PKC dependent pathways [42]. Sensory neuron sensitization occurs also through PGE_2_ effects downstream of PKA phosphorylation on voltage-gated Na_v_1.8 and Na_v_1.9 [43, 44] and voltage-gated Ca_v_3.2 [45], as well as P2X3 receptors [46, 47] and TRPV1 [48, 49]. Alternatively, downstream of PKA, PKCε may be activated, by means of Epac1 (the exchange protein directly activated by cAMP) [50]. Production of inflammatory prostaglandins through cyclooxygenase 1 and 2 is the mainstream target of anti-inflammatory and analgesic therapy. A new contribution to the action of ibuprofen might be TRPA1 inhibition by a metabolite [51]. A novel approach might be a more selective targeting of IP, EP_1_, EP_3_, and EP_4_. Such antagonists are available, for IP selexipag [52], for EP_1_ ONO-8130 [53], and for EP_4_, BGC20-1531 (phase I in 2009, not developed further) [54] and CR6086 (phase II started in 2017) [55]. However, it was argued that a simultaneous inhibition of these receptors might be required for analgesia, considering that combined but not separate IP and EP_4_ inhibition was effective [56]. EP_3_ knockout mice have a phenotype in a neuropathic pain model [57], but the antagonists have at least also other functions like antiaggregatory [58] and controlling micturesis [59].

### Cannabinoid Receptors

Despite a debate regarding psychotropics with at times a more political than medical agenda, this needs consideration. Regarding pain, there is a large body of literature [60]. A recent meta-analysis focusing on the clinical use of cannabinoids covered 28 trials with chronic pain assessment (2454 participants) and, despite limited effect size, revealed an overall greater pain relief with cannabinoids than with placebo [61]. There are two cannabinoid receptors, CB_1_ and CB_2_, both Gi-coupled GPCRs. As there is selective pharmacology, it is important whether CB_1_, CB_2_, or both receptors should be targeted. Although it was argued that peripheral CB_1_ is the main target for analgesia [62, 63], this is complicated by a regulation of these receptors in different pathophysiological conditions [64]. To limit central adverse effects, there are efforts towards the development of peripherally restricted CB_1_ agonists [65]. In addition to cannabinoid receptor agonism, positive allosteric modulation has the potential of a better side effect profile. ZCZ011 was analgesic in inflammatory and neuropathic pain models [66] and GAT211 acted against paclitaxel-induced neuropathy [67].

Further, an upregulation of endogenous ligands might be an option. Such endocannabinoids are anandamide (AEA) and 2-arachidonoylglycerol (2-AG). These are broken down by monoacylglycerol lipase (MAGL) and fatty acid amide hydrolase (FAAH), and their inhibition can elevate endocannabinoid levels. Spinal and supraspinal mechanisms contributed to analgesia from systemic FAAH inhibition, but this also has central side effects. A peripherally restricted FAAH inhibitor URB937 lacking such side effects caused CB_1_-mediated antinociceptive effects on inflammatory pain [68, 69]. Clinical development of FAAH inhibition failed for PF-04457845 tested against osteoarthritis [70], while BIA-102474 had severe side effects (interrupted phase I trial in 2016) [71]. Also, MAGL inhibition has been clinically tested: JZL184 raises 2-acylglycerol levels in the brain but has substantial central side effects [72]. FAAH and MAGL inhibition by PF-3845 and JZL184 is synergistic [73]. In particular, compounds with two targets have been sought. Dual FAAH inhibitor and TRPV1 antagonists AA-5-HT and OMDM-198 have analgesic potential, and might be promising in case the benefits of the dual action outweighs the disadvantages [74]. OMDM-198 had a neutral profile on body core temperature, this having been prohibitive for further progress of many TRPV1 antagonists [75]. Dual FAAH and COX2 inhibitor ARN2508 reduced intestinal inflammation [76]. Currently, there are several synthetic cannabinoids clinically used for nausea and vomiting, such as nabilone and dronabinol, that are also used off-label as pain treatment [77], with inhaled cannabinoids often used in conjunction with first-line analgesics. Current research is supportive of a role for cannabinoids in analgesia; however, additional carefully carried out trials are required.

### PAC_1_ Receptor

The pituitary adenylate cyclase–activating polypeptide (PACAP) type I receptor (PAC_1_R) is part of the vasoactive intestinal peptide/secretin/glucagon family of GPCRs and is also expressed in the peripheral nervous tissue [78]. Structure and ligand-binding have been clarified [79, 80]. Activation of PAC_1_R via PACAP or the agonist maxadilan were shown to induce and maintain nociceptive behaviors in rodents [81, 82], and attempts at blocking the receptor proved successful in attenuating formalin-induced pain [83]. PACAP38 is a potent inducer of migraine attacks with an increased selectivity towards PAC_1_R. Therefore, inhibition of the receptor is probed for migraine, e.g., by anti-PAC_1_R antibodies AMG 301 (phase II completed 2019) [84] or by the development of small-molecule antagonists [85]. The established antibiotic doxycycline in a subantimicrobial dose facilitates agonist-binding to PAC_1_R, which could allow to exploit an anti-inflammatory and neuroprotective potential [86].

### 5-HT_1_ Receptors

Serotonin receptors are classified into seven groups known as 5-HT_1_ to 5-HT_7_; all are GPCRs except 5-HT_3_ [87]. Members of this group have been targeted for the treatment of various types of painful conditions including neuropathic pain, migraine, and cluster headaches. The current theory of the pathophysiology of migraine highlights the role of the trigeminocervical complex under the effect of increased afferent activity. Additionally, the respective activity releases CGRP and PACAP from these nerves, leading to plasma extravasation [88]. Triptans have been a long-standing option for migraine treatment since the 1990s and are selective 5-HT_1B/1D_ receptor agonists, with some entities having affinity for the 5-HT_1F_ receptor [89]. It is assumed that at least part of the mechanism relies on activation of 5-HT_1_ receptors in the trigeminal ganglion, leading to reduced neuropeptide release, while CNS effects have not been excluded [90]. The main drawback of using triptans is their potential for vasoconstriction, due to activation of 5-HT_1B_ receptors. For selective 5HT_1F_ activation, a small but significant effect was found for lasmiditan, which was approved in 2019 [91].

Injection of serotonin into the muscle is painful in human volunteers [92]. Serotonin injection pain can be inhibited via the 5-HT_2B_ receptor in mice [93], and a role of this receptor was also demonstrated in neuropathic pain [94] while being assumed for migraine [95].

RS-127445 is a 5-HT_2B_ receptor antagonist which reduced allodynia after nerve injury [96]. So far, there are no clinical studies with this target. Drugs like selective serotonin reuptake inhibitors can reduce systemic levels of inflammatory cytokines (e.g., IL-1α, IL-6, IL-8, IL-12, IFN-γ) [97]. Within this category, the peripherally restricted N-methyl-citalopram has been patented and reported but not investigated for analgesic use [98].

### Proton-Sensitive GPCRs

There are many acid-modulated proteins, among which are four GPCRs, occurring in a variety of tissues. These include GPR4, GPR68, GPR132, and GPR65 [99]. The pH sensitivity and the expression patterns favor GPR68 (also OGR1) as a neuronal pH sensor, as it is found in about 29% of all sensory neurons and in 78% of putative nociceptors, indicated by the small-diameter neuron marker peripherin [100]. A role in inflammatory pain has been demonstrated [101, 102], and pharmacological inhibition resulted in attenuated colitis-associated pain [103]. Other potential uses to limit cellular acid-sensing, e.g., in cancer or cardiac ischemia [104, 105], require careful consideration of the usefulness of this approach for analgesia.

### Histamine

Histamine is mainly released by mast cells and activates four GPCRs (histamine receptors H_1_, H_2_, H_3_, and H_4_) involved in physiological and pathological processes. In terms of sensory physiology, histamine levels increase in inflammation, but the effects have been mostly associated with itch and allergy. There is evidence for H_3_ and H_4_ involvement in the modulation of nerve injury-induced neuropathic pain as well as in inflammatory pain models [106–108]. So far, there is only preclinical data to support the use of H_3_ and H_4_ antagonists for minimizing hypersensitivity [109]. The combination of peripheral and central effects might explain why H_3_ agonists as well as antagonists might be beneficial [110]. H_4_ receptor antagonists are further in development [111]. JNJ 7777120 inhibited a rabbit immune response to sheep erythrocytes [112], and adriforant (ZPL-3893787, discontinued after phase II) was anti-inflammatory in a phase IIa study for atopic dermatitis [113].

### C5a Complement Receptor

The complement system is an essential driver of innate immunity through opsonization and killing of bacteria. Three convergent activation pathways result in the formation of the membrane attack complex, through a precursor molecule called C5. The biologically active fragment C5a, as well as C3a, were shown to sensitize capsaicin-induced calcium transients in DRG neurons [114]. The C5a receptor mRNA codes for a GPCR in sensory neurons, which can induce thermal hyperalgesia requiring TRPV1 and NGF contribution. Moreover, C5a triggers arachidonic acid metabolism, and PGE_2_ synthesis [115], most likely through C5a receptor 1 activation. C5aR antagonism has been attempted with several strategies, among which a more recent one implies allosteric inhibition with DF2593A, proven to be effective in inflammatory pain models [116]. Anti-C5aR antibodies were effective at inhibiting the development of arthritis in rodents [117]. On the clinical side, selective C5aR inhibition by avacopan (CCX168, phase III completed, new drug application accepted) has been investigated in patients with vasculitis [118], the monoclonal antibody NNC0215-0384 (not further developed after phase I) in rheumatoid arthritis [119, 120], and anti-C5aR antibody avdoralimab has been lined up against inflammatory responses in the lung [121].

### Prokineticins

Prokineticin receptor 1 and 2 are GPCRs, activated by cytokine prokineticin 2 (Bv8). Both are expressed by neurons and have a role in pain [122, 123]. These receptors can sensitize ion channels, through kinases described below, including TRPV1 and acid-sensing ion channels [124]. PC1, an inhibitor prokineticin 1 [125], reduced neuropathic pain [126]. So far, this is in the preclinical stage; efforts to drug these targets have been summarized [127].

### Sensory Neuron Ion Channels

#### Sodium Channels

Voltage-gated sodium channels (Na_v_) play a major role in determining neuronal membrane excitability, which highlights their contribution to the genesis and maintenance of pain signals [128]. The mammalian Na_v_ family members have different expression patterns and possess heterogeneous electrophysiological properties [129, 130]. Na_v_ pharmacology is comprised essentially of blockers, some among the most well-established drugs for local anesthesia (lidocaine, bupivacaine), type I antiarrhythmics (mexiletine), or anticonvulsants (lamotrigine, carbamazepine, phenytoin) [131]. Inflammation has been shown to be associated with upregulation of Na_v_1.7 and Na_v_1.8 [132, 133], and Na_v_1.7 deletion abolished inflammatory pain responses in animal models [134]. Moreover, changes in activity, specifically decreased activation thresholds and ectopic discharges, most likely contribute to allodynia or hyperalgesia in neuropathic pain states [135], with certain lines of therapy including Na_v_ blockers. Therefore, it seems valuable to pursue selective pharmacological tools targeting Na_v_ subtypes. However, this development is ongoing for a considerable time and with substantial effort, indicating the difficulty to generate such subtype-specific and effective drugs [136–138]. Clinical trials have been exploring the utility of lidocaine patches in arthritis [139] and also alternatives to small-molecule antagonists, e.g., peptides (AM-6120, AM-8145, AM-0422) or Na_v_1.7 monoclonal antibodies [140]. Progress in development has been summarized [141].

#### Calcium Channels

There are ten calcium channels based on their alpha subunit, historically labeled by initials of substances acting on these, and more recently by genetic similarity. There are three families, the low-voltage-activated (T-type) Ca_V_3.x channels and the high-voltage activated calcium channels, which can be differentiated into dihydropyridine-sensitive (L-type) Ca_V_1.x and dihydropyridine-insensitive Ca_V_2.x. Antagonists of these voltage-sensitive channels are clinically used, in particular for targeting the cardiovascular system. Therefore, it has to be carefully considered whether there is an opportunity for systemic analgesic action [142]. Despite that, verapamil is a prophylactic option for cluster headache [143]. However, for migraine, L-type channel blockers and substances with a calcium-inhibitory component are considered [144]. Finally, several classic voltage-gated calcium channel inhibitors increase cytosolic calcium. It was shown that in the case of nifedipine, this is due to activation of TRPM3, TRPA1, and ionotropic glutamate receptors of the NMDA subtype [145]. A more novel approach might include antagonists of Ca_v_2.2 [146]. Based on the caveats mentioned above, it is interesting whether a weak calcium-channel inhibitory component might contribute to the overall action of an analgesic drug. Ca_v_3.1 has also emerged as an interesting target for analgesia in the context of neuropathic pain; Z944 (phase I in 2014) is a selective antagonist [147].

#### TRP Channels

Transient receptor potential (TRP) channels are grouped into 7 families, totaling 28 members [148]. The respective chapter is somewhat overrepresented as an example, but also as it reflects the core expertise of the authors. The scope of this review is broader; TRP channel-directed development of analgesics has been addressed in more focal reviews [149–151].

TRPV1 is the best-investigated pain target without a medically available antagonist. It is a well-established pain sensor, which is characterized by different modalities of activation. TRPV1 can be activated by temperatures over about 41 °C, chemical compounds (capsaicin, resiniferatoxin), and low pH and can be modulated downstream of a variety of bonafide inflammatory stimuli (e.g., bradykinin, prostaglandins), mostly through PKC-dependent pathways. Further stimulation can be mitigated through PKA phosphorylation [152], phosphatidyl-inositol-phosphates [153], and reactive oxygen species (ROS) effects, to a certain degree [154].

Nevertheless, TRPV1 is downstream of many inflammatory signaling pathways and pursuing strategies involving their inhibition might prove worthwhile. In the case of TRPV1, the consequence of locally applied capsaicin depends on the concentration and can lead to sensitization [155–158]. However, local capsaicin can also trigger desensitization of primary sensory afferents with analgesic outcomes, e.g., in the form of < 1% over-the-counter ointments indicated for the treatment of neuropathic and musculoskeletal pain [159]. Interestingly, doing this does not generate any meaningful changes in mechanical sensitivity. Transdermal 8% capsaicin patches are approved for postherpetic neuralgia-associated neuropathic pain [160]. Development of TRPV1 antagonists was highly pursued by the pharmaceutical industry, and one of the most important hurdles to be overcome seems to be minimizing off-target effects on body temperature. There are already pre-clinically confirmed modality-specific compounds, which largely leave body temperature untouched [161]. DWP-05195 and NEO-6860 have reached phase II trials (completed 2014 and 2016) for the treatment of neuropathic pain and osteoarthritis, respectively [162, 163], while tivanisiran, a small interference RNA inhibitor of TRPV1, failed its endpoints in a phase III trial for dry eye syndrome in 2019 [164]. Resolvin E1 has been shown to reduce substance P potentiation of TRPV1 in DRGs [165] and has reached phase I clinical trials in 2019 [166]; it might be useful for the treatment of rheumatoid arthritis, or other inflammatory conditions. A recent meta-analysis points towards differences in the role of rat and human TRPV1 thermoregulation, potentially a reason for the relatively poor translation of many antagonist effects in humans [167]. An interesting strategy might be heralded by the use of photoswitchable fatty acids, which could allow exposure of an optically defined area, as it is standard for photodynamic therapy. TRPV1 activation by photoswitchable capsaicin could allow highly localized neuronal ablation [168]. In summary, the development programs by most large pharmaceutical players have resulted in many substances, key side effects like self-inflicted burn injury and elevated body core temperature were slowly overcome, but the human trials so far had disappointing efficacy [169].

TRPA1 is even more promiscuous than TRPV1, as it is gated by a very wide range of stimuli including natural chemical compounds, drugs, calcium, voltage change [170], and UV radiation [171, 172], to name a few. In addition, TRPA1 has been shown to be sensitized by PKA [173] and prolonged agonist application [174, 175]. For a more comprehensive view over TRPA1 activation mechanisms as well as agonists and antagonists, see [148, 176, 177]. One of the main challenges in developing TRPA1 antagonists lies in a notable species difference, where in rodents, the effects may lead to underestimating potential clinical efficacy [178]. HC-030031 or A-967079 were the most promising antagonists, but have not been progressed to clinical testing. A phase II trial for neuropathic pain in diabetic neuropathy with GRC 17536 failed to meet the primary endpoint in 2014 [179]. Other attempts include ODM-108 [180], for neuropathic pain and CB-625 for acute surgical pain, both terminated in phase I [181]. Alternative strategies, such as drug repurposing for TRPA1, might see the use of desvenlafaxine, paliperidone, and febuxostat as TRPA1 blockers [182]. With the advent of potent TRPA1 agonists, a new human TRPA1 model can be used to test TRPA1 antagonists in humans [183]. Aside from pain, other diseases could benefit from TRPA1 inhibition [184]. However, similar to TRPV1, TRPA1 has been known for so long that the progress towards a usable analgesic is somewhat disappointing [185].

TRPM8 is viewed as the canonical cold transducer [186] and is also activated by menthol, which is routinely used for its analgesic properties in preparations to alleviate inflammatory pain in sports injuries or arthritis [187, 188]. There is an intriguing interplay between TRPM8 activity and inflammatory mediators. For example bradykinin, a proinflammatory mediator, exerts an inhibitory effect on TRPM8 activity, while TRPM8 activation by cold causes downregulation of proinflammatory TNFα [189]. TRPM8 antagonist RQ-00434739 reduced activity of afferents in bladder inflammation [190]. Systemic application of PF-05105679 successfully inhibited the cold pressor response of human subjects, but also led to unexpected hot sensations (discontinued after phase I completed in 2011) [191]. Also, AMG-333 was tested in phase I in 2013, but terminated thereafter without reported results [192]. Substantial progress has been made in the further development of agonists and antagonists [193].

TRPV4 is expressed in a large fraction of primary afferents and responds to, e.g., decrease of the osmolarity [194]. The knockout mice showed reduced mechanical and acidosis-induced pain [195]. Potent pharmacological tools have been developed [196]. Animal experiments suggested a pathophysiological role, e.g., in pulmonary, pancreatic, and bladder inflammation [197]. TRPV4 antagonist GSK2798745 has been reported as well-tolerated in a recent phase I trial [198]. The phase II trials for chronic cough [199] and LPS-induced alveolar barrier disruption [200] have been terminated due to a low probability of achieving the primary endpoint. In a third trial in 11 subjects with heart failure, alveolar diffusion capacity was not significantly changed [201]. Despite being beyond the peak of publication activity, it remains interesting whether this can be converted into human therapeutic benefit.

TRPV3 is mentioned here, despite reports of preferential expression in non-neuronal cells. Reduction of keratinocyte ATP release and with that the reduction of sensitization or activation was put forward as mode of action. TRPV3 antagonist GRC15300 was tested in a phase II clinical trial and terminated in 2013 after it failed to reduce neuropathic pain [202].

TRPV2 is heat sensitive. The higher thermal threshold compared with TRPV1 [203], which can be modulated [204], caused the speculation that this might be the sensor for noxious heat damage. The lack of an apparent phenotype of TRPV2 knockouts as well as TRPV1/TRPV2 double knockouts [205] limited interest for analgesic purpose.

TRPM2 might be an option to target pain [206], but its role in temperature sensing [207, 208] indicates that this might need to be overcome first to uncover analgesic potential.

TRPM3 activation evokes pain-related behavior in animals [209], and the contribution to heat pain in mice as part of a triad of redundancy in heat perception render this interesting [210]. In addition, TRPM3 is upregulated in inflammatory conditions, which increases the overlap with and the interaction with signaling of TRPV1 and TRPA1 [211]. TRPM3 is found in human sensory neurons [212], and concentrations of volatile anesthetics not exceeding the minimal alveolar concentration by far can act as TRPM3 antagonists [213].

#### Potassium Channels

Potassium channels are the largest and most diverse ion channel family with about 80 members [214]. As for the topics above, there are more focused reviews [215–217]. The potassium channels can be separated into four major groups, all present in primary afferent neurons. These are voltage-gated K^+^ channels (K_v_ families K_v_1–K_v_12), Ca^2+^-activated K^+^ channels (K_Ca_ families K_Ca_1–K_Ca_5), two-pore K^+^ channels (K_2P_ families K_2P_1–K_2P_17), and inwardly-rectifying K^+^ channels (K_ir_ families K_ir_1–K_ir_7).

The respective channels are discussed regarding their function in peripheral neurons [218]. Contribution to axonal conduction can reduce action potential frequency, partially also seen as action potential shape change. In particular K_v_2.1, K_v_3.4, K_v_9.1 and K_Ca_1.1 have been discussed to contribute to this in a frequency-dependent fashion, and the role of the T-junction might be therefore considered [219].

At least as interesting are channels which modulate spike initiation at the peripheral terminals, affecting resting membrane potential and action potential threshold. An antinociceptive decrease in primary afferent excitability can be brought about by opening potassium channels. This will hyperpolarize for a channel with an equilibrium potential below the resting membrane potential, but also increased potassium conductance without a change in resting membrane potential will stabilize against excitation. To this end, K_v_1 (K_v_1.1/K_v_1.2), K_v_7 (K_v_7.2/K_v_7.3), K_2P_ (TREK1, TREK2, TRAAK), K_Ca_1.1 are primarily considered, and this includes some controversy regarding expression in sensory neuron subtypes [215]. K_v_1.1/K_v_1.2 are mainly found in large non-nociceptive neurons [220]. K_v_7 channels, encoded by KCNQ1-5 historically named “M-channel” as it explained the observed non-inactivating potassium “M-current,” are a focus of pharmaceutical development since K_v_7 activation has demonstrated analgesic potential for inflammatory and neuropathic pain [221, 222]. Many of the respective studies used K_v_7 blocker XE-991 or K_v_7 activator retigabine [223, 224]. For currently used analgesics, a contribution of potassium channels to their analgesic action has to be considered, e.g., paracetamol metabolite NAPQI, meclofenamic acid, and diclofenac activate K_v_7.2 [225, 226]. Given homo- and heterotetramer formation, e.g., K_v_7.2/K_v_7.3 [227, 228], development of analgesics also depends on the generation of subtype-specific drugs. New K_v_7.2 agonists like ICA-27243 show antinociceptive effects and demonstrate active development in this area [229].

K_2P_ channel activators could also reduce excitability of afferent neurons [230]. K_2P_2.1 (TREK-1) activated by GI-530159 reduced excitability of rat sensory neurons [231] and antinociceptive effects of riluzole were ascribed to this channel [232]. Selective activators of K_2P_2.1 (TREK-1) and K_2P_10.1(TREK-2) allow probing these channels as analgesic targets [233]. Cloxyquin activates K_2P_18.1 (TRESK) [234]. Clinically established medications and endogenous substances are known to activate these targets [235], but pain-related studies are still rare and no clinical trials have been performed.

A search for substances tested in registered clinical trials concerning human pain modulation via potassium channels shows 4-aminopyridine, flupirtine, and maxipost. 4-aminopyridine, including the slow-release formulation fampridine, has shown some benefits in multiple sclerosis [236]. For the study testing 4-aminopyridine in Guillain-Barre syndrome patients [237] no results were published. The mechanism of action of 4-aminopyridine is unclear, since in addition to the K_v_1 inhibition, also a Ca_v_2.2 facilitation was demonstrated [238]. A change in pain was only a minor aspect [239]. Potassium channel opener flupirtine reduced human neuronal excitability [240].

Maxipost (BMS 204352) acts via multiple channels, including K_Ca_1.1, K_v_7.2, K_v_7.3, and K_v_7.4, but the anxiolytic effect demonstrates that this has a relevant central component [241, 242]. A headache-focused trial shows headache-induction by maxipost, indicating a role of BK_Ca_ channels in headache pathophysiology [243]. The same group has also reported that opening of ATP-sensitive potassium channels was associated with occurrence of headache [244].

#### Chloride Channels

Intraneuronal chloride homeostasis is maintained by multiple players, and the outline of a topical review [245] has been used here to focus on analgesic potential. Chloride channels are comprised of GABAA and glycine receptors, calcium-activated chloride channels (CaCC), ClC family of chloride channels and transporters, cystic fibrosis transmembrane conductance regulator (CFTR), volume-regulated anion channels (VRAC), and maxi-anion channels. All of these adjust intracellular chloride levels, impacting on excitability of sensory neurons. The equilibrium potential of chloride depends on the intracellular Cl^−^ concentration, such that in case of a high concentration, channel opening can cause depolarization [246, 247]. The hypothesis that neuronal Cl^−^ accumulation can amplify Cl^−^ efflux and, consequently, alter sensory signaling has been supported by targeting NKCC1 (Na^+^-K^+^-2 Cl^−^ transporter). The respective knockout animals had reduced thermal responses [248] and NKCC1 inhibitor bumetanide reduced inflammatory pain in the formalin test [249]. Inflammatory mediators bradykinin, PGE_2_, NGF, and ATP inhibit KCC2 (K^+^–Cl^−^ cotransporter), a main player in Cl^−^ outflow, and facilitate chloride import through NKCC1, which as net effect increases excitability [250].

Calcium-activated chloride channels include anoctamins, which consist of 10 channels. For the first member, anoctamin 1, expression was predominantly found in small DRG neurons [251], and a contribution to excitability of neurons has been demonstrated [252]. Human anoctamin 1 can be inhibited by MONNA with an IC50 of about 1 μM [253] and animal data point towards an analgesic effect [254], but this needs to be further scrutinized, also helped by new antagonists [255]. Functions of anoctamin 1 in other systems, e.g., epithelial secretion, have to be considered for side effects [256]. Bestrophins (Best1–4) are also chloride channels, with expression in intermediate-sized sensory neurons [257]. Members can be activated by slight intracellular calcium elevation, which would allow an amplification of minor signals. A role in a neuropathic pain model for bestrophin 1 has been described [258], but the pharmacological tools to advance this are still limited. The voltage-gated chloride channel CIC3 was found in small sensory neurons, and upregulation reduced excitability [259]. TMEM206 has recently been described as a proton-activated chloride channel, but it appears predominantly expressed in the central nervous system [260]. Cystic fibrosis transmembrane conductance regulator is a chloride channel mainly known due to the consequence of the recessive genotype. It is found in neurons, peripheral and central [261], and contributes to neuronal ATP release [262]. The lack of pain phenotypes in CFTR patients questions a role of this channel in disease pathophysiology.

Volume-regulated anion channels respond to osmotic changes [263]. LRRC8A, an essential member, is found in DRG neurons [264]. Whether it can be targeted to control chloride levels remains to be elucidated. Maxi-anion channels are widely expressed and can be recruited by cellular stress [265]. This includes inflammatory conditions, which were shown to regulate SLCO2A1 [266]. So far, pain as part of a syndrome has only been described in case reports.

GABAA receptors are found on sensory neurons, and local GABA injection increases excitability in mice and humans [267, 268]. An antinociceptive effect could be expected from GABAA antagonists, but systemic application of substances penetrating the blood–brain barrier have a convulsant effect [269]. Peripherally restricted GABAA receptor antagonists, e.g., bicuculline methiodide, were so far not reported to inhibit pain, which might suggest that there is no tonic GABA release contributing to baseline excitability.

#### Acid-Activated Ion Channels

Acidosis is one of the main features of inflammation, with protons being sourced through cell lysis along with other mediators and by relative ischemia. Tissue acidosis leads to nociception, by direct gating or through sensitization to other agonists [270]. Canonically, proton-gated acid-sensing ion channels (ASICs) [271] form a group of voltage-insensitive sodium channels, with four members (ASIC1–4) [272]. This feature is also present in a variety of other channels, including TRPV1, TRPV4, TRPC4, TRPC5, TRPP2, P2X purinoceptors, inward rectifier K^+^ channels, voltage-activated K^+^ channels, L-type Ca2^+^ channels, HCN channels, gap junction channels and Cl^−^ channels [273], and can be sensitized by inflammatory mediators [274, 275]. In humans, low pH-induced pain in the range of acidosis occurrence is mediated by TRPV1 [276], whether other targets contribute substantially in inflammatory conditions is currently unknown. The most likely target for ASIC analgesia in inflammation remains ASIC3, with substantial expression in the peripheral nervous system [277, 278]. In a human trial, ASIC blocker PPC-5650 had no relevant effect on mechanical, thermal, electrical, and chemical stimulations of the esophagus [279].

#### Mechanoreceptors

Mechanotransduction is fundamental to the environmental interaction of living organisms. Sensing of mechanical stimuli, innocuous or noxious, is strongly affected by inflammation, as most inflammatory conditions are accompanied by protective hyperalgesia [280, 281] and allodynia [282]. Mechanosensation, mechanonociception and the differentiation between these two sensory modalities represent a longstanding interest in neuroscience research. Competing theories regarding how they function are the labeled line theory of somatosensation and the population-coding model [283, 284]. More and more light is shed on the molecular pathways of mechanosensitivity. Key players include Piezo2 [285], Na_v_1.1 [286], neuronal S1P receptor S1P_3_ [287], TRPV4 [288], and epithelial Na^+^ channel (ENaC) [289]. In addition, several cellular microcompartments such as primary cilia, caveolae, or integrins can sense the stiffness of the extracellular matrix [290, 291].

Sensory neurons respond to sphingosine 1 phosphate (S1P), involving TRP ion channels [292], but the contribution to neuropathic pain also involves non-neuronal cells, e.g., through the S1P receptor 1 in astrocytes [293]. Sphingosine 1 phosphate contributes to inflammation [294], but this signaling is also involved in acute mechanonociception by S1P_3_ [287]. S1P_3_ knockout mice showed attenuated mechanical pain and inflammatory thermal hypersensitivity. There are two FDA-approved S1P_1_ antagonists, namely fingolimod and siponimod. Fingolimod stimulates S1P receptor internalization and has so far been therapeutically used against multiple sclerosis relapse [295]. Fingolimod reduces neuropathic pain, however, by reducing central sensitization in the dorsal horn [296]. A further trial exploring its utility in neuropathic pain is underway [297].

An interesting argument has been made in the case of TRPV4, an established sensor of shear stress and change in osmolarity, referring to a role in transducing forces in cell contacts [290]. TRPV4 deficient mice seem to be protected when being faced with acute lung injury [298] and pharmacological inhibition leads to similar results [299], reducing neutrophil infiltration and levels of inflammatory mediators such as IL-6.

In the long-enigmatic mechanotransduction, the late discovery of the unusual Piezo cation channels stands out [300]. The rodent and the human form of Piezo2 respond to low mechanical forces [285, 301, 302]. Bradykinin has been shown to increase Piezo2 mechanosensitive currents via PKC and PKA pathways in sensory neurons [303]. However, the primary function of the Piezo channels seems to be touch, and there is less contribution to stronger pain-inducing stimuli. Neurons lacking Piezo2 responded robustly to noxious pinch [304]. When touch becomes allodynic in inflammation, Piezo2 seems to contribute, and human subjects with a loss of function mutation report less sensitivity to capsaicin-induced neurogenic inflammation [305]. A relief from inflammatory pain can only be hypothesized, but this still awaits the availability of an antagonist. For Piezo1, a negative interaction with Piezo2 has been demonstrated [306].

#### HCN Channels

Hyperpolarization-activated cyclic nucleotide-gated channels (HCN1–4) are nonselective cation channels conducting both sodium and potassium ions [307]. HCN upregulation in the trigeminal ganglion has been demonstrated for inflammation at the dura mater [308] and HCN2 knockout mice exhibit diminished heat hyperalgesia in inflammatory pain models [309]. HCN3 has been largely excluded from having any relevant implications in inflammatory pain [310]. HCN2 and HCN4 are modulated by cAMP and PKA, which might be selectively targeted [311]. Ivabradine, a peripherally restricted general HCN blocker, provided analgesia in inflammatory and neuropathic pain mouse models [312]. However, in healthy volunteers, ivabradine lacked impressive analgesic properties but decreased heart rate, indicating that more HCN2-specific blockers might be required [313]. To this end, compounds selective towards HCN1/2, such as MEL55A, might yield better results on this front [314].

#### Purinergic Signaling

Purinergic receptors are divided into three main groups: P1 with members A_1_, A_2A_, A_2B_, A_3_ is G protein-coupled, the P2 family has been divided into the P2X ion channels P2X1–P2X7 and the P2Y G protein-coupled receptors with 8 members. Purinergic signaling is an important driver of inflammation as ATP is released into extracellular space by inflammatory cells [315, 316], and iontophoretic ATP application into human skin caused a concentration-dependent pain response [317]. An in-depth review for purinergic signaling in pain has been provided [318].

The role of P2X receptors in pain has been reviewed [319]. A role in nociception has been shown for the purinergic receptors P2X2 and P2X3, which can form heterodimers [320]. P2X3 receptors are attractive analgesic targets due to their exclusive expression on sensory neurons [321]. A-317491 reduced inflammatory and neuropathic pain in rats [322], with further improvement of pharmacokinetics and a reduction of thermal and mechanical hyperalgesia in an endometriosis pain model [323]. P2X3 antagonist gefapixant (AF-219) reduced chronic cough in a phase II trial [324]. AF-219 has advanced to phase III, BLU-5937 to phase II, and the progress in development has been recently summarized [325]. P2X4 [326] and P2X7 [327] are also involved in pain, but their primary mechanism works through microglia. As purinergic GPCRs have not been given a separate paragraph, it should be mentioned that neuronal P2Y1 activation has been shown to elicit sensitization through TRPV1 [328]. With the release of ATP, also ADP, AMP and adenosine are generated. Targeting adenosine A1 receptors with GR79236X failed to reduce pain due to a third molar extraction [329].

### Enzymes in Sensory Neurons

#### Protein Kinase A

Many inflammatory mediators’ signaling pathways converge to an increase in intracellular levels of cAMP (e.g., prostaglandins, bradykinin). Among downstream targets of cAMP, contributing to neuronal hypersensitivity, is the AMP-dependent protein kinase (PKA), itself a sensitizer of pain-transducing targets, including TRPV1 [330], TRPV4 [331], and TRPA1 [173], as well as Na_v_1.8 [332] and Na_v_1.7 [333]. PKA activity in inflammatory hyperalgesia plays an important role in its onset as well as in maintenance, as PKA inhibitors have shown reduction of hyperalgesia in animal models [334, 335]. PKA has four regulatory subunits, of which R1β has a predominantly neuronal expression pattern, and the respective knockout mice have a phenotype in inflammatory pain [336]. However, there are so far only inhibitors for multiple protein kinases (AGC inhibitors), with effects also on PKA for cancer treatment [337, 338], but not PKA subtype-specific antagonists.

An alternative pathway for cAMP involves the exchange proteins directly activated by cAMP (Epac), which function to catalyze the exchange of GDP to GTP on small Rap proteins, leading eventually to PKCε-and mitogen-activated protein kinase activation [339]. Epac function seems to be intertwined with alterations in nociception; for example, Epac activation leads to TRPV1 sensitization, an effect blocked by inhibiting downstream targets PKCα and PKCε [340]. Epac-selective cAMP analogue 8-pCPT has been shown to sensitize mechanically evoked Piezo2-mediated currents in DRG neurons and induce mechanical allodynia through Epac1 [341]. More recently, Epac inhibitor ESI-09 was used to suppress chemotherapy-induced pain [342] or inflammatory pain [343], but so far, Epac modulators have been limited to preclinical research [344].

#### Protein Kinase C

Protein kinase C (PKC) is important for regulating several neuronal functions, with sensitizing effects on many ion channels involved in nociception, such as TRPV1 [345, 346], ASICs [347, 348], and Na_v_s [332, 349]. Since PKC activity is sensitive to intracellular levels of calcium, it is also subject to modulation by inflammatory mediators acting through the PLC pathway, many of which are discussed below. Among the 16 PKC isoforms, PKCε seems to be critical for the development of acute inflammation, as shown with genetic deletion and pharmacological inhibition [350]. So far, PKC inhibitors, as many other kinase inhibitors, were mostly developed regarding their antitumor activity. Preclinical and clinical efforts in development were summarized [351]. The furthest progress was made for aprinocarsen which failed in phase III for lung cancer [352]. Subtype-specific knockouts have demonstrated less neuropathic pain, e.g., for PKCγ [353]. The importance of PKC subtypes for pain has been discussed [354]. The PKCε inhibitor KAI-1678 (studies completed in 2011) was well tolerated, but had no beneficial effect for postherpetic neuralgia and postoperative orthopedic pain [355, 356]. Overall, for PKC, the challenge remains to find a sufficiently nociception-specific approach, which is equally valid for all ubiquitous kinases.

Anchoring of PKC, PKA, and also phosphatase 3A to their targets occurs through adapter proteins, for these enzymes in particular through the A-kinase anchoring proteins (AKAP); this serves to facilitate sensitization driven by bradykinin and PGE_2_ in certain channels, e.g., TRPV1 [357] and TRPA1 [358]. These anchoring proteins have specific interfaces to their phosphorylated targets, which could avoid the side effects of the kinases’ many targets, but allow to inhibit a specific interaction [359]. However, this has so far also remained elusive.

#### Mitogen-Activated Protein Kinases

MAPKs are a diverse family of serine–threonine kinases which include extracellular signal-regulated kinase (ERK), p38, and c-Jun N-terminal kinase (JNK) [360]. These signaling cascades are involved in cell proliferation, and respective inhibitors have largely been evaluated or used for cancer. However, these kinases are also involved in sensitization, and their analgesic potential, directly on neurons or indirectly, e.g., reducing tumor progression, needs to be discussed [361]. In DRGs, MAPKs are activated under cellular stress conditions and proinflammatory cytokines exposure, e.g., NGF, TNFα, and thermal stimulation [362–364]. MAPK inhibitors have been extensively used for alleviating allodynia and hyperalgesia in animal models of inflammatory pain. Below are recent preclinical and clinical developments pertaining to this complex pathway. ERK inhibition with U0126 resulted in improved thermal hyperalgesia after capsaicin injection [365], normalized indices of mechanical allodynia and heat hyperalgesia [366], and alleviated chemotherapy-induced neuropathy [367]. Compounds in late stages of development or already on the market include selumetinib, which reduced pain in children with inoperable plexiform neurofibromas, again the relative importance of desensitization *versus* slowing of disease progression being unclear [368]. P38 inhibitors appeared to be effective in rheumatoid arthritis (PH-797804, discontinued after phase II) [369], the inflammasome was inhibited by CDD-450 (ATI-450, phase II, new ongoing study) [370, 371], and pain after nerve injury was reduced by dilmapimod (SB-681323, phase II trials completed several years ago) [372]. JNK contributes to inflammatory pain, also via non-neuronal cells in the DRG [362, 373]. Whether MAPK inhibitors prove to be useful as analgesics for patients without need for antitumor therapy or even within this group remains an open question.

#### Src

Src is a ubiquitously expressed tyrosine kinase, with important roles in several signaling pathways, including cell growth, division, and survival [374]. It is also strongly linked with a number of targets of inflammatory mediators (e.g., NGF) triggering the PI3K-PKB-Src pathway, and leading to TRPV1 upregulation [375]. TRPM8 function also depends on the phosphorylation state, which is regulated by Src [376]. Similarly, the role of Src in inflammation and neuropathic pain has been investigated in conjecture to the NMDA receptor complex, whose function it also enhances [377]. Using a Src inhibitor peptide, the authors suppressed both inflammation and nerve injury-induced pain, leaving other sensory functions intact. Non-specific tyrosine kinase inhibitors which also target Src, e.g., dasatinib, imatinib, are used therapeutically against several types of cancer, where they also inhibit cancer-induced pain [378], but have not been investigated so far in terms of analgesia.

### Interleukin Receptors, at Least Also on Sensory Neurons

Interleukins are numbered, which obfuscates that these are clustered in superfamilies, labeled by a prominent or early member. Here, according to the review structure, these are primarily sorted based on whether they act on a receptor on sensory neurons. An overview of cytokine targets in pain has been provided [379, 380].

#### IL-1

The IL-1 receptor has been found on sensory neurons [381], although there is also conflicting evidence, where the receptor was only on DRG-supplying vessels [382]. IL-1β-increased excitability of isolated sensory neurons should settle this issue [383]. The IL-1 superfamily consists mostly of proinflammatory mediators, key members are IL-1α, IL-1β, IL-18, IL-33, IL-36α, IL-36β, and IL-36γ, acting on a series of own receptors [384]. There are also antagonists to these respective receptors, e.g., IL-1Ra, IL-36Ra, which can belong to other superfamilies. IL-1α has been labeled as a dual function cytokine with a nuclear localization sequence in its precursor region, in addition to it targeting its cell membrane receptor [385]. IL-1β potently induces hyperalgesia, e.g., in skin or in joints [386, 387]. The cascade is complex and involves PGE_2_, substance P, nitric oxide, and endothelial adhesion molecules. Vice versa, IL-1β occurs also downstream upon injection of well-established inflammatory agents [388]. That review also discussed IL-1β involvement in glia–neuron interaction, assuming a role in neuromodulation in persistent pain states.

In clinical studies, IL-1α and IL-1β administration leads to fever and generalized systemic inflammation, parenting strategies for disrupting the respective pathway [389, 390]. There is no doubt about the merit of targeting IL-1 signaling in pathophysiology. There are antibodies against anti-IL-1α and anti-IL-1β and against the IL-1 receptor [391], and with the first mention of therapeutic antibodies, a review of this treatment strategy is provided [392, 393]. A monoclonal antibody against IL-1α reduced pain in refractory cancer patients and improved quality of life [394]. A strategy to scavenge IL-1β before it binds to its target is through soluble receptor decoys rilonacept, gevokizumab, and canakinumab [395]. IL-1 receptors can be blocked by recombinant antibodies; anakinra was the first one to be approved in 2001 for rheumatoid arthritis and its use has been expanded to other autoinflammatory diseases [391].

A novel strategy serving this purpose is in the form of a chimeric IL-1α/IL-1β structure, inactive until conversion to the active form at sites of inflammation [396], potentially reducing the risk of infection. It should be mentioned that the IL-1R1 receptor antibody AMG108 (development discontinued) has not shown substantial effects in clinical trials [397]. All of these entities are clinically investigated in trials spanning a broad spectrum of diseases, and it remains to be scrutinized more thoroughly if they can be helpful in inflammation-induced hyperalgesia [391].

#### IL-6

IL-6 receptor presence in sensory neurons was shown by mRNA [398] and on a protein level, using glycoprotein 130-like immunoreactivity [399]. TLR activation in monocytes and macrophages due to extracellular damage induces mRNA-transcription of IL-6 through the NF-κB pathway, along with other potent pro-inflammatory cytokines, in a similar fashion to TNFα and IL-1β [400]. PGE_2_, e.g., produced by macrophages, also stimulates IL-6 release [401, 402]. Classic IL-6 signaling works by forming a hexameric complex comprising two molecules each of IL-6, the membrane bound or the soluble form of the IL-6 receptor and the signaling receptor component gp130 [403–406]. Signaling is mediated through the ubiquitous gp130 via the JAK/STAT transduction pathway. The role of IL-6 varies according to the organ it is produced in; in hepatocytes, it induces acute-phase proteins, such as the C-reactive protein [407], while in bone tissue, it is tightly linked with osteoclastogenesis and bone remodeling [408]. IL-6 is elevated in several models of peripheral nerve injury [409–411], and targeting IL-6 attenuates neuropathic pain [412]. Moreover, IL-6 induced calcium transients in a third of cultured rat DRG neurons, and longer exposure increased NK1 receptor immunoreactivity and substance P induced calcium transients [399]. Satellite glia cells were found to also exhibit increased levels of IL-6 and its receptor upon constriction injuries of the sciatic nerve, suggesting involvement in sensitization [413].

Therapeutic approaches are mainly based on monoclonal antibodies, targeting IL-6 by sirukumab and siltuximab [414, 415] or the IL-6 receptor by tocilizumab [403]. So far, diseases responsive to tocilizumab seem to be characterized by long-term IL-6 action (Castleman disease, rheumatoid arthritis); the respective efforts have been summarized [416, 417]. Tocilizumab showed promising results for discogenic low back pain [418]. Several studies also pointed towards differential effects of IL-6 *versus* IL-6 receptor antibodies, suggesting alternative signaling pathways not involving the IL-6 receptor [419].

IL-6 superfamily members with an association to inflammation and pain are leukemia inhibitory factor (LIF), oncostatin M, which are found on sensory neurons, with IL-27 and IL-35 discussed in the “Immune Reaction” section.

#### Leukemia Inhibitory Factor

In contrast to the proinflammatory function of LIF, there is also evidence of an anti-inflammatory and even neuroprotective role [420], with both points of view summarized elsewhere [421]. LIF receptor mRNA and protein have been identified in murine DRG neurons [422, 423]. Nevertheless, testing of LIF monoclonal antibodies seems to be directed towards other fields than nociception, e.g., infertility [424]. After less successful interventions on chemotherapy-induced neuropathic pain [425], it has shown promise for pancreatic cancer [426].

#### Oncostatin M

Oncostatin M signals through gp130 and the oncostatin M–specific subunit beta, which is also expressed in sensory neurons, colocalized with TRPV1 and the P2X3 purinergic receptor [427]. One of its main roles seems to be in the development of nociceptors, as genetic ablation of oncostatin M resulted in reduced sensitivity to noxious mechanical and thermal stimuli [428]. This is in line with evidence of oncostatin M–dependent heat-induced hypersensitivity via TRPV1 sensitization [429]. A wider use has been demonstrated with inflammatory heart failure [430]. A trial with oncostatin M monoclonal antibody GSK2330811 has completed phase I (phase II ongoing) [431]. Therapeutic options for a more general anti-inflammatory use can still be conceptualized.

#### IL-4 and IL-13

IL-4 receptors are found in both mouse and human sensory neurons and elicit functional responses [432]. A new concept is a fusion protein of two anti-inflammatory interleukins; the IL4–10 fusion protein has shown an analgesic effect *in vivo* in a model of osteoarthritis [433, 434]. Related to IL-4 is IL-13, which is secreted by immune cells and changes the phenotype of local macrophages towards an anti-inflammatory phenotype. Peripherally administered IL-13 reduced allodynia in a neuropathic pain model [435]. Targeting IL-4 and IL-13 with the monoclonal antibody dupilumab has proven clinically effective against inflammatory conditions, for atopic dermatitis patients [436, 437] as well as in chronic rhinosinusitis [438, 439]. This is in line with a similar effect of Janus kinase inhibitor delgocitinib (phase II studies in 2020), which reduces IL-13, IL-4, and IL-31 levels [440].

#### IL-17

IL-17 receptors have been found on sensory neurons, can sensitize mechanical nociception, and prolonged exposure changes the expression of pain-related targets, e.g., TRPV4 [441, 442]. IL-17 family members IL-17A–IL-17F are produced by a T helper cell subset called Th17. These cytokines signal through a set of heterodimeric receptors, comprised of an IL-17RA chain, and a second chain, serving ligand specificity [443]. The canonical activation leads into NF-kB and MAPK pathway activation, including ERK, p38, JNK, and CCAAT-enhancer-binding proteins (C/EBP) [443, 444]. IL-17A might have the potential to mediate mechanical hyperalgesia through receptors on nociceptive neurons [442]. Recent successful therapeutic approaches have mainly targeted the progression of psoriasis inflammation, e.g., by IL-17A-blocking antibodies secukinumab and ixekizumab and the IL-17RA-targeting antibody brodalumab [445–448]. A small-scale study showed a decline in pain ratings in psoriatic patients with subclinical joint inflammation [449].

#### IL-31 and IL-33

These interleukins were reported in conjunction with allergic diseases and itch [432]. The receptors for IL-31 and IL-33 are on sensory neurons, and the mediators can directly excite mouse DRG neurons [450, 451]. IL-31 was found to signal through the brain natriuretic peptide and is also involved in the antinociceptive as well as pruritogenic actions of morphine [452]. Hypertrophic scars can be associated with itch and pain [453], and the IL-31 signaling in this tissue was upregulated [454]. Nemolizumab, an antibody against the IL-31 receptor reduced pruritus in atopic dermatitis [455]. In psoriatic patients, lesioned skin had lower pain thresholds and elevated IL-33 expression compared with control skin [456]. There is substantial preclinical evidence supporting that this target could be exploited [457], and human trials using monoclonal antibodies against IL-33 or ST2 are in early stages [458–460].

#### IL-2

The IL-2 receptor was found to be expressed in the DRGs, in particular on small and medium-sized neurons, and its activation increased pain thresholds [461]. However, the recombinant IL-2 variant, aldesleukin is prescribed for metastatic kidney cancer or metastatic melanoma, indicating that analgesia is not the primary therapeutic effect.

### Neurotrophin Receptors

Neurotrophins, such as NGF and BDNF, are induced in inflammatory conditions [462, 463]. They work via the tyrosine kinase-coupled receptors of the Trk family and, depending on the concentration and duration of exposure, can elicit hyperalgesic effects [464]. The receptors for NGF and other neurotrophins are found on sensory neurons [465, 466], which is also the reason why NGF is frequently added to primary afferent neuron cultures.

Injection of NGF produces acute hyperalgesia, most likely through trkA signaling. In the long term, NGF causes sensitization through many effects, including mobilization to the membrane [467] and regulation of expression [468], axonal transport [469], axonal growth [470], and neuronal survival [471]. NGF increases expression of receptors involved in generating painful sensations, such as ASICs, sodium channels, and bradykinin receptors, and by release of neuropeptides [472–475]. The proinflammatory actions of NGF can be inhibited, with a reduction of allodynia and thermal hyperalgesia demonstrated more than 20 years ago [476]. Since then, therapeutic options have increased, including scavenging of NGF by antibodies, inhibition of NGF binding to trkA, and the inhibition of trkA function [477]. Despite intermediate setbacks from arthritis therapy, the monoclonal antibodies against NGF are an important contribution of anti-inflammatory options [478], e.g., for arthritis, but also for low back pain [479]. BDNF receptors were found in the sensory neurons and an antibody directed against BDNF reduced bone cancer pain [480]. The central effects, also useful for other diseases, make this less of a pain target. The therapeutic options for GDNF, BDNF and NGF have been recently summarized [481].

### Other Targets on Sensory Neurons

#### TLR4

Toll-like receptors (TLRs) are a family of well-conserved transmembrane receptors that initiate immune cell responses by recognition of pathogen-associated molecular patterns [482]. Their expression extends from immune cells to glial cells and DRG neurons [483–485]. TLR ligands have been shown to induce upregulation of several inflammatory mediators in DRG cultures [486, 487] and particularly TLR4 has emerged as an interesting target for inflammatory pain [488]. It was hypothesized that TLR4 might facilitate conversion towards chronic pain states [489] and also help maintain it [490]. TLR4 as a target to control neurogenic inflammation has been discussed [491]. Monoclonal anti-TLR4 antibody NI-0101 (phase II completed in 2018) did not show efficacy for rheumatoid arthritis [492]. TLR4 antagonist TAK-242 has antinociceptive effects in rodent neuropathic pain, low back pain and disc degeneration, experimental pancreatitis, and ischemia–reperfusion-induced pain [493–496]. Suppressor of cytokine signaling 3 (SOCS3) allows an anti-inflammatory intervention through TLR4 [497]. This could be enhanced by paeoniflorin, but immunomodulation by this compound has consequences, including the inhibition of the inflammasome and TRPV1 [498], and the potential of suppressor of cytokine signaling 3 cannot be judged with the available results.

#### Prolactin Receptor

The prolactin receptor, a type I cytokine receptor, is activated by prolactin, a polypeptide hormone with major roles in lactation and reproduction among serving regulatory effects in growth, development, and immune functions [499]. Prolactin production has been described for several tissues, including the pituitary gland, CNS, immune cells, placenta, and mammary glands [500, 501]. The main downstream effector pathway of prolactin/prolactin receptor signaling is the JAK/STAT pathway, and MAPK is also activated [502]. Nevertheless, the prolactin receptor is intriguing in the context of nociception due to its prolactin-dependent sensitization predominantly in females, which also worked in isolated sensory neurons of females [503, 504]. More specifically, it has been hypothesized that the female sex hormone estradiol regulates translation of prolactin receptor mRNA. Alternative splicing generates a long and a short isoform of the prolactin receptor [503], and the latter seems to regulate neuronal excitability via TRPA1, TRPV1, and TRPM8 channels and modulate opioid-induced hyperalgesia [505, 506]. Pharmacological tools are available, e.g., competitive antagonist Delta1-9-G129R-hPRL [507] or the monoclonal antibody (LFA102, phase I in 2012, continued as X213 in 2016), which has been evaluated against prostate and breast cancer [508, 509]. Delta1-9-G129R-hPRL could antagonize prolactin-induced sensitization of ASICs [510]. A consequence of prolactin receptor signaling inhibition could be the dysfunction of pituitary homeostasis, e.g., pituitary hyperplasia; therefore, it is critical to consider potential side effects of prolactin receptor antagonists when attempting to establish analgesic control [511].

#### ClC-6

This is an electrogenic 2Cl^−^/H^+^ exchanger with expression in afferent neurons, localized intracellularly to late endosomes [512]. ClC-6 knockout mice tolerated noxious heat longer, which might render this an interesting target to inhibit once an antagonist has been discovered.

#### Programmed Cell Death 1

The programmed cell death 1 receptor (PD-1) belongs to the immunoglobulin superfamily and is expressed by T cells [513], and also in the periphery, e.g., on epithelial and endothelial cells. The ligand PD-L1 acts as an immune suppressor and is expressed by several types of tumors, including melanomas [514]. However, the receptor and its ligand PD-L1 are also found on DRGs, and PD-L1 was shown to reduce excitability through PD-1 in rodent and human primary sensory neurons [514, 515]. Conversely, blocking the PD-L1/PD-1 pathway elicited spontaneous pain. PD-1 was required for opioid antinociception, demonstrated in knockout mice, but also with the clinically used monoclonal anti-PD-1 antibody nivolumab [516]. An association between the soluble ligand sPD-1 and pain in cancer patients has been reported [517]. Several immune checkpoints inhibitors involving the PD1/PD-L1 pathway are approved for cancer treatment [518]; however, an overview of pain scores in treated patients is not yet available. Adverse neuromuscular effects should be mentioned [519]. Nevertheless, before this can be used as antinociceptive or opioid-enhancing, the challenge remains to disentangle immunosuppressive action from the antinociceptive one.

## Mediators and Antagonists Acting on Sensory Neurons

Mediators and targets of these play overlapping roles in inflammation and inflammatory disorders. For sorting in this review, molecules with many targets were sorted in “Mediators and Antagonists Acting on Sensory Neurons,” when the mediator appeared more essential than a particular of its respective receptors on sensory neurons. However, in case no such receptor is known, the target is mentioned in “Neurotrophin Receptors.” Mediators can trigger the release of other mediators, generating a stimulus pattern. Some mediators can function bimodally, serving as proinflammatory or anti-inflammatory, depending on the context. Mediators which are not direct effectors of the nociceptive neurons can still be essential components of an inflammatory cascade. Therefore, targeting these in a disease-modifying approach might provide means to disrupt pathophysiology, and justifies to expect an analgesic potential and therefore discussion here.

### Lipid Mediators

These are diverse and include e.g., prostaglandins, leukotrienes, hydroperoxyeicosatetraenoic acids (HPETEs), hydroxyeicosatetraenoic acids (HETEs), and epoxyeicosatrienoic acids (EETs). The generating pathways as well as options to intervene were well summarized [520, 521].

Prostaglandins originate from membrane phospholipids, which under the catalytic activity of PLA2 and PLA1 generate arachidonic acid and lysophospholipids. Arachidonic acid is further processed by cyclooxygenase 1, regulating baseline levels of prostaglandins, and cyclooxygenase 2 inducing prostaglandin production during inflammation. Additionally, in the brain, arachidonic acid is liberated from 2-arachidonoylglycerol through endocannabinoid hydrolysis [522]. As the concentration of the important mediators critically depends on a rate-limiting enzyme activity, the latter has been successfully targeted. Cyclooxygenases are widely expressed in many tissues [523]; the resulting mediators are primarily not generated by the sensory neurons and depend on the involved cells [524]. The respective drugs are most established [41, 523] and among the most frequently used analgesics [525]. Lipidomic screens demonstrated that the available drugs have a differential effect on the resulting mediator patterns [520]. There are still efforts for further development [526].

Lipoxygenases convert arachidonic acid to leukotrienes, HPETEs and HETEs. An important role in leukotriene biosynthesis is held by the 5-LOX-activating protein (FLAP); as the name suggests, it serves to enhance enzyme activity, doing so through binding to arachidonic acid and presenting 5-LOX to it [527]. Potentially targetable sites along the leukotriene metabolic pathway therefore include FLAP, 5-LOX, LTA4, and LTB4 receptors [520]. FLAP as a target in inflammatory diseases has been discussed [528]. Some of the more advanced FLAP inhibitors include licofelone (phase III in 2010, no new drug application) for osteoarthritis [529], and veliflapon (phase III in 2006, no new drug application) for cardiovascular pathology [530]. Other attempts at developing FLAP inhibitors mainly oriented them towards asthma treatment with compounds such as MK-0591 (quiflapon, discontinued after phase II in 2015) [531], or GSK2190915 (several phase II trials, completed many years ago) [532]. 5-LOX inhibitor development has also been pursued, outputting compounds such as flavocoxid (discontinued after phase I) [533] and atreleuton (VIA-2291, discontinued after phase III) [534]. Diflapolin, a dual inhibitor of 5-LOX and soluble epoxide hydrolase, emerged as a promising anti-inflammatory tool [535]. LTB4, an end product of the LOX signaling pathway has been involved in inflammation [536] and has been shown to generate hyperalgesia in an intracutaneous injection model in humans [537]. Interestingly, its receptors, GPCRs BLT1 and BLT2, seem to have antagonizing functions, with BLT2 activation being pronociceptive and BLT1 activation being antinociceptive, e.g., converging on TRPV1 [538]. Although pharmacological tools exist for these targets, it remains to be seen how clinical development unfolds.

### Lysophospholipids

Lysophospholipids share one acyl chain, which remains after cleavage of phosphatidic acid by phospholipase A1 and A2 or by cleavage of acyl-lysophosphatidic acid or lysophosphatidylcholine by lysophospholipase D (autotaxin). Lysophosphatidic acid is a more general term describing several variations of the molecule, depending on the degree of saturation and the length of the acyl chain [539]. LPA modulates the function of members of most ion channel families [540]. In addition, there are also phospholipid receptors, in particular on cells of the immune system [541]. LPA has been shown to be involved in the genesis of neuropathic pain through direct intrathecal injection [542]. In humans, it seems that LPA levels correlate with the severity of pain symptoms in patients with various types of neuropathies [543]. LPA-generating autotaxin levels correlate with the intensity of pruritus symptoms in patients with cholestatic itch [544]. In contrast to previous findings of direct TRP channel activation [545], LPA18:1 more substantially activates satellite glial cells and Schwann cells, suggesting an indirect neuromodulatory action [546]. LPA receptor antagonists are under development, and being tested for a number of pathologies, with analgesia not as the primary focus [539, 547, 548].

### Tumor Necrosis Factor Superfamily

TNF inhibitors are clinically used for more than 30 years. This therapeutic group has become medically important and is therefore mentioned here not as a novel approach, but as a benchmark for the potency of other approaches discussed below.

The TNF superfamily is comprised of 19 ligands and 29 receptors [549] of which probably TNFα is the most important and extensively studied due to its strong proinflammatory effects [550]. TNFα acts pronociceptive; unilateral injection of TNFα caused a bilateral TRPV1-dependent pain, which involves PKC, prostaglandin E2, and IL-1β [551]. Pain-related behavior induced by the PKC activator PMA was reduced in mice treated by an anti-TNFα antibody [552]. TNFα is cleaved from a membrane-bound precursor by the metalloproteinase TNFα-converting enzyme (TACE). During the immune response, a variety of cells release TNFα, including activated macrophages, dendritic cells, monocytes, NK cells, CD4+ T cells, CD8+ T cells, microglia, and astrocytes [553, 554]. TNFα acts on TNF receptor 1 and TNF receptor 2 [555]. These receptors are found on various cell types, but it should be mentioned that they are upregulated in the DRG during inflammation [550]. In rats, intrathecal knockdown of the TNF receptor 1 decreased inflammatory hyperalgesia [556]. Based on the strong preclinical effects, antibodies against TNFα have been developed and are on the market since 1988 [557]. The primary indication is autoimmune disease, including rheumatoid arthritis, psoriasis, Crohn’s disease, ulcerative colitis [558, 559]. Clinical trials investigating peripheral pain using TNFα inhibitors had a promising start [560] but, overall, provided mixed results. Infliximab was not better than placebo in patients with disc herniation-induced sciatica [561] and etanercept showing varying improvements in acute lumbosacral radiculopathy [560, 562, 563]. Finally, adverse reactions at the injection site should be mentioned [564].

### Trypsin

Trypsin belongs to the PA clan superfamily of serine proteases. Pathophysiology can be differentiated between the pancreas and other sites. For acute pancreatitis, an antinociceptive effect of inhibition of serine proteases was shown [565], and protease-activated receptor 2 (PAR2) contributes to this [566]. In line with this, PAR2 also contributes to pain in pancreatic cancer [567].

In tumors, PAR2 is triggered as a result of increased levels of proteolytic activity [568]. PAR2 contributes to inflammatory pain [569], in particular to mechanical-induced pain [570]. For trypsin, also an activation of vagal neurons through PAR1 has been demonstrated [571]. In the peripheral nervous system, the endogenous serine proteinase inhibitor serpin3A inhibits leukocyte elastase, and inhibition of leukocyte elastase reduced neuropathic pain in mice [572].

### Substance P

Substance P was identified in the early 1930s as a vasodilating substance and structurally characterized approximately 40 years later as a undecapeptide [573, 574]. It serves as the ligand for the neurokinin receptor 1 with roles in, among others, pain and inflammation [575, 576]. Substance P is released from a series of immune cells [577] but also from terminals of primary sensory neurons in response to stimulation [578]. Upon release, or after local injection, substance P has been shown to increase inflammatory cytokine levels whereas a neurokinin 1 receptor antagonist was effective in reducing sensitization in an incisional model [579]. Intracellular signaling following neurokinin 1 receptor activation involves PLC-dependent intracellular calcium elevations, and also PKC and PKA activation [580, 581]. In terms of clinical use for neurokinin 1 receptor antagonists, they are attractive for targeting inflammation, but have been oriented towards reducing chemotherapy-induced nausea and vomiting, e.g., aprepitant [582]. Neurokinin 1 receptor antagonists have been considered for headache [583], but results have been disappointing [584, 585]. Also for other types of pain, in contrast to preclinical results, human data showed no analgesia for neurokinin 1 receptor inhibition [586]. A potential benefit of a more complex inhibition of multiple neurokinin receptors is unclear, or whether targeting this pathway can serve to potentiate analgesia in certain circumstances.

### CGRP

Calcitonin gene-related peptide is a neuropeptide expressed in a fraction of sensory neurons and released upon their activation in a calcium-dependent manner [587]. Not too many new classes of analgesics have become available in the past, but targeting CGRP is one of these, with clear benefit for migraine patients [588]. Fremanezumab, galcanezumab, eptinezumab target CGRP, while erenumab targets the CGRP receptor.

Activity in the trigeminal system is thought to reflect headache. CGRP stimulated trigeminal activity [589] and CGRP antagonist olcegepant reduced trigeminal activity [590]. Mechanisms underlying sex-dependent differences in animals and humans are unclear [591]. Despite effective phase III trials for small molecule antagonists, poorly published concerns regarding liver toxicity effect have halted further development. Animal models support a role of CGRP in liver disease [592]. The proof of concept was put to success with monoclonal antibodies against CGRP and the CGRP receptor [588]. Given their success and independence from triptan responders, there is new incentive for further consideration and development of small-molecule antagonists [593]. This paragraph was placed in the mediator section, as the functional role of CGRP receptors on neurons is unclear. CGRP does not excite the terminals [594], is found on Schwann cells and on central but not peripheral axons [595], and the site of action is also proximal to the ganglion [596], assumably postsynaptic.

### Acidosis

Local pH decrease is a robust follow-up of the onset of inflammation, resulting from immune cell infiltration, increased demand in oxygen and energy, and accelerated glycolysis and acidic molecule secretion, such as lactic acid [597]. In addition, local phagocytic bursts result in a dramatic increase in local proton concentrations [598]. This in turn has been shown to stimulate proinflammatory factor secretion, such as TNFα and IL-1β [599, 600]. Low pH has been shown repeatedly to be an activator of sensory neurons [601, 602]. Several structures can in principle sense low pH, including acid-sensing ion channels (ASICs), K_2P_ channels, the four proton-sensitive GPCRs discussed above, and TRPV1 [270, 603]. Peptide isolation from the black mamba venom has also revealed an interesting new group of molecules, mambalgins, targeting acid sensors with analgesic effects [604, 605]. Recently, it has been reinforced that in humans, TRPV1 is the main contributor to mild and pathophysiologically relevant acidosis-induced pain in the skin [276], which supports using modality-specific TRPV1 antagonists as a new tool for inflammatory pain.

### ADAMTS

ADAMTS abbreviates for “a disintegrin and metalloproteinase with thrombospondin motifs.” In contrast to ADAMS, which are mainly cell membrane proteins, ADAMTS are secreted enzymes [606]. This family is comprised of 19 members of which ADAMTS-4 (aggrecanase-1) uses chondroitin sulfate hyaluronan-binding proteoglycans as a substrate, including aggrecan [607]. ADAMTS-4 and ADAMTS-5 are proteases with important developmental roles [608], associated with inflammatory disorders, in particular osteoarthritis, where cartilage degradation results in joint pain [609]. Aggrecan is attached to the surface of chondrocytes, and with it the bulk of chondroitin sulfate, a critical water absorbing component of the cartilaginous structure [610]. The cleaving process mediated by ADAMTS-4 and ADAMTS-5 releases the chondroitin sulfate–modified C-terminus from the chondrocytes into the synovium, and inhibitors could prevent osteoarthritis cartilage loss. In this regard, several substances with antagonizing effects have been investigated, including disease-modifying anti-rheumatic drugs, nonsteroidal anti-inflammatory drugs, and statins [608]. Tissue inhibitor of metalloproteinase TIMP-3 could serve as a prototype for biological treatments [611, 612]. In summary, these targets appear to reduce pain primarily through disease modification. For ADAMTS-13, despite some search hits, pain occurs also only disease-associated and the literature does not suggest analgesic potential.

### Glutamate Carboxypeptidase II

Glutamate production, to which this enzyme contributes, is a basis of this important neurotransmitter, and might be an alternative to inhibition of receptors for glutamate [613]. Mice deficient of this enzyme had less neuropathic pain [614], and an orally available antagonist for this enzyme reduced neuropathic pain [615].

### Amino Acid Metabolism

Amino acids are typically not classified as mediators, but are nevertheless discussed here. Tumors and other fast-dividing tissues depend on metabolites, including glucose and amino acids. Therefore, nutrient deprivation of these has been tested to limit tumor growth [616]. Amino acids of interest are glutamine, serine, methionine, asparagine, and arginine [617]. Therefore, key enzymes of the respective metabolic reactions have been considered targets, and this might be extended to the control of pain. Caloric restriction has been associated with a reduction of pain in rodents [618, 619]. A change in amino acid composition has also been useful in low back pain patients [620], although a normalization of lowered amino acid levels was observed. A dietary intervention also improved chronic pancreatitis, again with a mixed effect on plasma amino acid levels [621].

Attempts to target individual amino acid metabolism for pain included glutaminase inhibition. However, antagonist CB-839 failed to reduce cancer-induced bone pain, which might be due to more metabolic flexibility of the tumor than expected [622]. Another option would be targeting transmembrane amino acid transporters. It remains to be determined whether a metabolic intervention allows to reduce pain with an acceptable level of adverse effects.

### IL-36

IL-36α, IL-36β, and IL-36γ have been outlined as relevant promoters for skin psoriasis, psoriatic arthritis, and rheumatoid arthritis [623]. The anti-inflammatory cytokine interleukin-36Ra is reduced in psoriasis [624] and anti-IL-36 receptor antibodies reduced tissue inflammation in a psoriasis mouse model [625]. IL-38, the most recent addition to the IL-1 superfamily, is an anti-inflammatory cytokine, acting through inhibition of the IL-36 receptor [626], and reduces IL-1β, IL-6, IL-8, IL-1α, and TNF-α [623, 627, 628].

## Immune Reaction

The general function of the immune system and the many and differentiated approaches for anti-inflammatory intervention are beyond the scope of this effort and are regularly reviewed. The aim of this last chapter is to point the reader to recent literature on this topic, in particular for matters considered of relevance to pain.

### Cytokines as Part of the Immune Reaction

Therapeutic options to target cytokines in inflammatory diseases have been discussed [629].

#### IL-18 and IL-37

IL-18, first described as “interferon gamma-inducing factor,” is structurally close to IL-1β. It is nevertheless functionally distinct [630], e.g., it does not induce PGE_2_ production [631]. The IL-18 receptor is primarily found in immune cells [632]. In rats, intrathecal IL-18 application reduced pain withdrawal thresholds and IL-18 scavenging by an antibody or a binding protein alleviated nerve injury-induced hypersensitivity [633, 634]. The respective mechanism appears to involve spinal microglia. IL-18-binding protein is a constitutively secreted protein, with higher affinity for IL-18 than the IL-18Rα and is supposed to act as a down-regulator of Th1 immune responses through IL-18 binding and reduction of IFNγ induction [635]. Tadekinig alfa is a recombinant human IL-18-binding protein that has been under clinical scrutiny for the treatment of adult-onset Still’s disease [636] and could hold promise for providing analgesia in inflammatory diseases with imbalance between IL-18 and the IL-18 binding protein. IL-37 is an endogenous suppressor of proinflammatory cytokine secretion, reducing IL-1β, IL-8, IL-1α, and TNFα in patients with systemic inflammatory diseases [637]. IL-37 in association with the IL-18-binding protein inhibits the IL-18 receptor alpha [638]; in addition, SMAD family member 3 is discussed as a target [639]. For anti-inflammatory interleukins, application of a recombinant form might be an option and has been used for other interleukins [640]. Recombinant IL-37 is available and might serve as a viable strategy for reducing inflammation [641]. However, until the moment of writing, this has not been clinically investigated.

#### Colony-Stimulating Factors

Granulocyte colony-stimulating factor (G-CSF) and granulocyte–macrophage colony-stimulating factor (GM-CSF) have been shown to act directly on the respective receptors on DRG neurons, causing sensitization [642]. G-CSF is the primary driver of neutrophil differentiation. Binding of G-CSF to its receptor induces signal transduction through STAT3, PI3K, and ERK [643]. Recombinant G-CSF filgrastim and lenograstim are clinically used for neutrophil regeneration after chemotherapy or myeloablation [644]. Adverse effects include inflammation, swelling, pain, and stinging; the most common adverse effect of pegfilgrastim and lenograstim is bone pain [645, 646]. In addition, there seems to be a role for microglia in G-CSF-driven neuronal hyperexcitability [647]. G-CSF monoclonal antibodies have entered clinical trials [648] and show some promise for the treatment of persistent pain following inflammation resolution.

GM-CSF is principally expressed in myeloid precursors, mature monocytes, and granulocytes [649]. The respective receptor is found on myeloid cells, in particular monocytes and granulocytes [650]. GM-CSF signals through JAK2/STAT5a/b in addition to PI3K, RAS/MAPK, and NF-κB [651]. Recombinant GM-CSF accelerates wound healing [652], with an obvious implication of inhibition of this mechanism. Studies using GM-CSF knockout mice and monoclonal antibodies against GM-CSF have shown that the cytokine is key in the development of osteoarthritis and rheumatoid arthritis–associated pain [653]. Moreover, silencing GM-CSF signaling in a model of peripheral nerve ligation induced analgesia, supporting a role in neuropathic pain upon intrathecal and not peripheral delivery of anti-GM-CSF receptor antibodies [654]. Clinical studies involving mavrilimumab were promising for targeting this pathway in rheumatoid arthritis [655] and could be explored further as a tool for providing analgesia in inflammatory conditions.

Macrophage colony-stimulating factor (M-CSF or CSF1) expression is increased in DRG neurons following peripheral nerve injury with the M-CSF receptor mRNA being induced in spinal microglia [656]. Recombinant M-CSF given intrathecally led to mechanical allodynia, while blocking its receptor suppressed mechanical allodynia after SNI [657].

#### IL-12

Interleukin 12 is produced in monocytes, macrophages, and dendritic cells [658]. IL-12 generates mechanical but not thermal hyperalgesia upon intraplantar injection into the rat hindpaw, with the endothelin B receptor being reported to mediate this action [659]. Endothelin B receptors are expressed on DRG neurons; however, endothelin 1 and synthetic agonist IRL-1620 decrease the excitability of DRG neurons [660], which seems contradictory to the IL-12 effect being mediated through endothelin B receptors. Only one of two heteromeric IL-12 subunits has been identified in the mouse DRG and was surprisingly downregulated after sciatic nerve damage [661]. Nevertheless, patients receiving recombinant IL-12 infusion as therapy for various types of cancer, reported painful symptoms, such as arthralgias [662] or headaches and abdominal pain [663], allowing to consider anti-IL-12 as a concept.

#### IL-27 and IL-35

IL-27 and IL-35 are structurally different (heterodimeric cytokines) to the original members, however still considered a part of the IL-6 superfamily. Genetic ablation of IL-27 was shown to deprive mice of an innate IL-10-dependent antinociceptive reaction in a nerve injury model [664]. IL-27 acts anti-inflammatory, limiting thermal and mechanical sensation, as mice lacking IL-27 or its receptor spontaneously showed chronic pain-like hypersensitivity, with reversal of the behaviors upon recombinant IL-27 injection [665]. Anti-inflammatory cytokine IL-35 was antinociceptive in an autoimmune encephalomyelitis pain model [666]. The anti-inflammatory properties of IL-35 were also shown in a model of diabetic neuropathy, associated with JNK downregulation [667] and neuroprotective microglial M2 polarization [668].

#### IL-23

Interleukin 23 is mainly released from dermal dendritic cells in skin psoriasis models. This stimulates a γδ-T cell subset, triggering IL-17 and IL-22 secretion [669], and can lead to itch, pain, and discomfort. Interestingly, a TRPV1- and Na_v_1.8-positive subpopulation of sensory neurons was required for the IL-23 production of dermal dendritic cells, the signaling pathway from nociceptors to dermal dendritic cells is unclear [670].

#### Interferons

Receptors for type I interferons IFN-α and IFN-β are expressed in small and medium-sized DRG neurons. The activation of these receptors increases excitability and produces mechanical pain in mice [671].

The receptor for type II interferon gamma (IFNγ) has also been reported on DRG neurons [672]. IFNγ is produced by T cells and other immune cells [673] and has a proinflammatory role [674] acting on microglia [675]. IFNγ is medically used for osteopetrosis and chronic granulomatous disease, the antibody against IFNγ emapalumab for the treatment of haemophagocytic lymphohistiocytosis [676]. In summary, this does not indicate a relevant analgesic potential for interferons.

#### IL-10

Interleukin 10 is considered an important anti-inflammatory cytokine, signaling through the IL-10 receptor which is found primarily on leukocytes [677]; an expression on sensory neurons has not been reported. Pathways include STAT1, STAT3, PI3K, and p38 MAPK [678, 679], inhibiting the synthesis of proinflammatory cytokines such as IFN-γ, IL-2, IL-3, TNFα, and GM-CSF.

Recombinant IL-10 has been tested as anti-inflammatory treatment in clinical trials, but results were heterogeneous, e.g., disappointing for rheumatoid arthritis, but promising for psoriasis [680]. However, also proinflammatory actions were observed [681], including flu-like symptoms [682]. Given the many studies, a general anti-inflammatory and thereby analgesic role seems unlikely.

### Chemokines

Chemokines are a family of cytokines with around 50 members. These small secreted proteins act on GPCRs and have important roles in leukocyte chemotaxis [683]. Some of the receptors are also expressed at the level of primary afferents [684]. A function of these is neuron to glia signaling, e.g., sensory neuron CX3CL1 induces microglial activation via CX3CR1, as well as glia to neuron signaling, e.g., CCL2 and CXCL1 from spinal astrocytes acting on neuronal CCR2 and CXCR2. Moreover, it has been shown that a series of chemokines, many of which secreted by resident leukocytes, can exert direct excitatory effects on primary sensory neurons, and also lead to the release of substance P [685]. This might account for some of the increased pain sensitivity in a variety of inflammatory conditions. The challenge for effective analgesic therapeutics to emerge is targeting proalgesic effects without undermining useful immune function, hence the relative paucity of clinical trials exploring chemokine receptor antagonists’ analgesia.

### Free Radicals in Inflammation

The framework describing the role of redox reactions in cellular homeostasis and pathological processes is more recently known as “the redox code” [686] or similarly the “reactive species interactome” [687]. Reactive oxygen species are well-documented, with one of the most important being superoxide. Produced mainly in the mitochondria, by interaction of leak electrons with oxygen or through NAD^+^ oxidation, via xanthine oxidase [688] or even as a result of COX or LOX function, superoxide is reduced to the subsequent ROS species, hydrogen peroxide and the hydroxyl radical. The latter is especially unstable and reacts readily, with membrane lipids fueling lipid peroxidation processes and even with DNA [689]. The role of ROS in the early stages of inflammation or tissue injury is well established, as they are produced during the respiratory burst in phagocytic cells [690]. More so, ROS production is a result of most antitumor drugs and the main mechanism by which photodynamic therapy works [691]. The intense pain generated by photodynamic therapy is explained by a photosensitizing effect on primary sensory neurons, namely the subpopulations expressing TRPA1 and TRPV1, which have also been shown to be directly gated by ROS [171, 172, 692]. With regard to reactive nitrogen species, nitric oxide matches one of the smallest molecular sizes with an extremely important role in vascular biology, acting as an endogenous vasodilator [693]. NO is produced by nitric oxide synthases. Coded by separate genes on different chromosomes, nitric oxide synthases are either constitutive (endothelial, neuronal eNOS, nNOS) or inducible (iNOS). The first two produce transient pico- to nanomolar concentrations of NO and are dependent on intracellular calcium levels, whereas iNOS is calcium-independent and results in long-lasting micromolar concentrations of NO [694]. Some NO results from swallowed nitrite (NO_2_^−^), or from nitrites in the UVA or blue light–exposed skin [695]. In sensory neurons, NO plays a very important role in modulating pain sensitivity during inflammation [696, 697], as confirmed by the analgesic effect of NO synthesis inhibitors [698]. Downstream of NO, soluble guanylate cyclase activation takes effect, leading to increases in cGMP [699], also in central spinal cord neurons [700]. Direct infusion of NO causes pain in humans [701] and TRPA1 and TRPV1 have been shown to be NO sensors [702]. Reactive sulfur species are less well characterized than the others, potentially due to less stability in experimental conditions. Interestingly, there is an emerging interest for interaction with RNS (NO/H_2_S crosstalk) and the formation of sulfur/nitrogen reactive species (nitrosopersulfide, SSNO) [687] and HNO in the presence of hydrogen sulfide [703, 704]. Further, NO can disrupt cysteine bonds and form S-nitrosylated residues, with structural consequences for (membrane) proteins [705]. Inhibiting or scavenging reactive species has not been a convincing strategy to combat pain and pathophysiology. Inhibiting the target of these reactive species, in particular TRPA1, might be more promising.

### NLRP3

NLRP3 (NALP3) is a Nod-like receptor subset with an inflammasome-forming pattern recognition receptor of the innate immune system, a multimeric protein complex with NLRP3 as the sensor component, alongside adaptor protein ASC and the effector caspase 1, and is representative of a major inflammatory pathway [706, 707]. Downstream effects of NLRP3 activation include increased levels of proinflammatory mediators, spearheaded by IL-1β, eventually accounting for hypersensitivity [708]. Moreover, this particular inflammasome is triggered in conditions such as gout, rheumatoid arthritis, and fibromyalgia [709]. The clinical progress with several antagonists for the NLRP3 pathway has been reviewed [710]. Targeting the NLRP3 inflammasome has been attempted with varying degrees of success. Tranilast, approved in 1982, has been found to inhibit the assembly of NLRP3 inflammasome [711]. However, tranilast can induce cystitis [712], which might be partial agonistic through TRPA1 [713]. MCC950 [714–716] and dapansutrile have reached phase II [717] and could be a promising new approach.

## Conclusion

This work aimed at summarizing the status quo of preclinical and clinical research of well-established targets for analgesia as well as showcasing novel, potentially addressable elements of a multitude of signaling pathways involved in nociception, the majority of which, of inflammatory nature. One must always consider far-reaching repercussions, especially pertaining to immune system function and pathology-appropriate treatment scenarios. However, too many new approaches fail due to translational hurdles, adverse effects, lack of efficacy, but also regulations. A positive outlook is that most of the presented strategies involve specificity towards one or several targets (e.g., monoclonal antibodies), some already in different phases of clinical trials.

## Electronic Supplementary Material

ESM 1 (PDF 1224 kb)

## Abbreviations

2-AG: 2-Arachidonoylglycerol
5-HT: Serotonin
5-HT_1–7_: Serotonin receptors 1–7
ADAMTS: A disintegrin and metalloproteinase with thrombospondin motifs
ADP: Adenosine diphosphate
AEA: Anandamide
AMP: Adenosine monophosphate
ASIC: Proton-gated acid-sensing ion channel
ATP: Adenosine triphosphate
BDNF: Brain-derived neurotrophic factor
BPS: British Pharmacological Society
C/EBPs: CCAAT-enhancer-binding proteins
CaCC: Calcium-activated chloride channels
Ca_v_: Voltage-gated calcium channels
CB_1–2_: Cannabinoid receptors
CC: C-C motif chemokine
CFTR: Cystic fibrosis transmembrane conductance regulator
CGRP: Calcitonin gene-related peptide
ClC: Chloride channel family
COX: Cyclooxygenase
CX3C: C-X3-C motif chemokine
DDC: Dermal dendritic cells
DP1: PGD2 receptor
DRG: Dorsal root ganglia
EETs: Epoxyeicosatrienoic acids
ENaC: Epithelial Na^+^ channel
EP_1–4_: PGE receptors
Epac: Exchange factor directly activated by cAMP
ERK: Extracellular signal-regulated kinase
FAAH: Fatty acid amide hydrolase
FDA: Food and Drug Administration
FLAP: 5-LOX-activating protein
FP: PGF receptor
G-CSF: Granulocyte colony-stimulating factor
GDNF: Glial cell line–derived neurotrophic factor
GDP: Guanosine diphosphate
GM-CSF: Granulocyte–macrophage colony-stimulating factor
GPCR: G protein–coupled receptor
GTP: Guanosine triphosphate
H_1–4_: Histamine receptors 1–4
HCN_1–4_: Hyperpolarization-activated cyclic nucleotide-gated channels 1–4
HETEs: hydroxyeicosatetraenoic acids
HGCN: Hyperbolic graph convolutional neural network
HPETEs: Hydroperoxyeicosatetraenoic acids
IFN: Interferon
IL: Interleukins
IP: PGI receptor
IUPHAR: International Union of Basic and Clinical Pharmacology
JNK: c-Jun N-terminal kinase
KCC2: K^+^–Cl^−^ cotransporter
K_ir_: Inwardly rectifying K^+^ channels
LIF: Leukemia inhibitory factor
LOX: Lipoxygenase
LPA: Lysophosphatidic acid
LTB4: Leukotriene B4
MAGL: Monoacylglycerol lipase
MAPK: Mitogen-activated protein kinase
M-CSF: Macrophage colony-stimulating factor
Na_v_: Voltage-gated sodium channels
NF-kB: Nuclear factor kappa-light-chain-enhancer of activated B cells
NGF: Nerve growth factor
NK1: Neurokinin 1
NKCC1: Na^+^–K^+^–2Cl^−^ transporter
NLRP3: NOD-like receptor family subset
NO: Nitric oxide
NOS: Nitric oxide synthase
OSM: Oncostatin M
PAC1R: PACAP type I receptor
PACAP: Pituitary adenylate cyclase–activating polypeptide
PAR: Protease-activated receptor
PD-1: Programmed cell death 1 receptor
PD-L1: Programmed cell death 1 ligand 1
PGD_2_: Prostaglandin D2
PGE: Prostaglandin E
PGF: Prostaglandin F
PGI: Prostacyclin
PI3K: Phosphatidylinositol 3-kinase
PKA: Protein kinase A
PKC: Protein kinase C
PLA: Phospholipase
PLC: Phospholipase C
RGD: Rat Genome Database
ROS: Reactive oxygen species
S1P: Sphingosine-1-phosphate
S1PR: Sphingosine-1-phosphate receptor
SOCS3: Suppressor of cytokine signaling 3
STAT: Signal transducer and activator of transcription
TACE: TNFα converting enzyme
TIMP: Tissue inhibitor of metalloproteinase
TNFα: Tumor necrosis factor alpha
TP: TXA receptor
trkA: Neurotrophic receptor tyrosine kinase 1
TRP: Transient receptor potential (channels)
TXA: Thromboxane A
VRAC: Volume-regulated anion channels
